# The role of IL-2 in type 2 immunity

**DOI:** 10.3389/fimmu.2025.1622187

**Published:** 2025-09-10

**Authors:** Naoya Tatsumi, Yosuke Kumamoto

**Affiliations:** ^1^ Center for Immunity and Inflammation, Rutgers New Jersey Medical School, Newark, NJ, United States; ^2^ Department of Pathology, Immunology and Laboratory Medicine, Rutgers New Jersey Medical School, Newark, NJ, United States

**Keywords:** IL-2 (interleukin-2), T helper type 2 (Th2), ILC2 - group 2 innate lymphoid cell, dendritic cell (DC), type 2 immunity

## Abstract

Type 2 immune responses are essential for protective immunity against helminth parasites and for promoting tissue repair, but they can also drive allergic inflammation. T helper type 2 (Th2) cells and group 2 innate lymphoid cells (ILC2s) are key drivers of these responses, producing hallmark type 2 cytokines such as IL-4, IL-5, IL-9 and/or IL-13. While IL-2 has long been recognized as a T cell growth factor, emerging evidence reveals its central role in shaping Th2 cell fate and function. This review focuses on recent studies on how the availability of IL-2 is regulated *in vivo* for inducing Th2 cells. We also discuss the role of IL-2 in activating ILC2s and thereby linking innate and adaptive immune system in the context of type 2 immunity. Together, the studies discussed here highlight the role of IL-2 as a spatially and functionally dynamic coordinator of type 2 immunity.

## 1 Introduction

Type 2 immunity plays a central role in host defense against helminth parasites and in tissue repair, but it also contributes to pathogenesis in allergic diseases and asthma. This immune axis is driven by both adaptive and innate components, most notably T helper type 2 (Th2) cells and type 2 innate lymphocytes (ILC2s), which together mediate responses through the production of type 2 cytokines including interleukin (IL)-4, IL-5, IL-9 and/or IL-13. IL-2 is often considered as a driver of “neutral” proliferation of CD4^+^ T cells that promotes expansion of antigen-specific CD4^+^ T cells without skewing them to a particular effector subset, but recent studies underscore its role in activating both Th2 and ILC2s in the context of type 2 immunity. Importantly, growing evidence indicates that the availability of IL-2 *in vivo* is tightly controlled both quantitatively and spatially. This review focuses on recent advances in our understanding of the role of IL-2 in type 2 immunity, particularly focusing on how its availability is regulated in the activation of Th2 cells and ILC2s.

## 2 Role of IL-2 in Th2 cell differentiation

### 2.1 IL-2 in CD4^+^ T cell differentiation

Originally identified as a T cell growth factor, IL-2 plays a central role in proliferation and maintenance of T cells (1, 2). Upon TCR stimulation, the activated T cell upregulates the expression of IL-2 as well as CD25 (IL-2Rα), the high affinity subunit of IL-2 receptor (IL-2R). Together with CD122 (IL-2Rβ) and the common γ chain (CD132/IL-2Rγ), which are expressed in naïve T cells, CD25 forms the high affinity IL-2R heterotrimer complex in activated T cells (3). The IL-2R signals mainly through phosphorylated STAT5 (pSTAT5) and further upregulates CD25, forming a positive feedback loop for CD25 expression (4, 5). In the case of CD4^+^ T cells, this upregulation of CD25 is followed by their differentiation into effector T helper cell subsets such as Th1, Th2, Th17 and T follicular helper (Tfh) cells.

While these initial findings established the role of IL-2 as an autocrine/paracrine survival and growth factor for activated T cells, the IL-2R signaling suppresses IL-2 production (5, 6), potentially leading to differentiation of IL-2-producer and IL-2-responder subsets. In fact, more recent studies highlight the role of IL-2 as a key regulator of CD4^+^ T cell differentiation that suppresses Tfh cell differentiation while facilitating non-Tfh effector fate and inhibits Th17 cell differentiation (7–21). In addition, CD4^+^ T regulatory cells (Treg) naturally express high levels of CD25 and rely heavily on IL-2 for their survival, serving as an “IL-2 sponge” that tightly regulates the local concentration of IL-2 and limits the availability of IL-2 in other T cells (22–24).

### 2.2 IL-2 in Th2 cell differentiation

#### 2.2.1 IL-2 in Th2 cell differentiation

The role of IL-2 in Th2 cell differentiation through the downstream pSTAT5 has been extensively studied *in vitro*. During the early stage of Th2 cell fate specification, the IL-2-induced pSTAT5 promotes the Th2 fate by directly binding to the *Il4ra* locus early after activation and later to the *Il4* and *Gata3* loci to induce their expression, which then triggers the IL-4-IL-4Rα-STAT6-GATA3 positive feedback loop (13, 25, 26). Although STAT5 and GATA3 target distinct cis-regulatory elements (e.g., STAT5 at HSII/HSIII and GATA3 at CNS-1/VA of the *Il4* gene), their co-expression synergizes to maximize IL-4 production, thereby reinforcing Th2 differentiation and identity (12, 13, 27–29). In parallel, the transcription factor c-Maf contributes to this process not only by directly transactivating the *Il4* promoter (30), but also by enhancing CD25 expression in an IL-4-independent manner (11), thereby sensitizing cells to IL-2 and sustaining STAT5 activation. Accordingly, c-Maf-deficient Th2 cells exhibit delayed CD25 expression and reduced pSTAT5 expression in response to stimulation with anti-CD3/CD28 monoclonal antibodies (mAbs) (11).

In support of the critical role of the IL-2–pSTAT5 axis in Th2 cell differentiation, CD4^+^ T cells in both *Stat5a^−/−^* and *Stat5b^−/−^* mice exhibit impaired Th2 cell differentiation when stimulated *in vitro*, though *Stat5a^−/−^* CD4^+^ T cells show specific reduction in Th2 cells while *Stat5b^−/−^* CD4^+^ T cells show more global reduction in both Th1 and Th2 cells (10). Likewise, in mice sensitized intranasally with house dust mite (HDM), CRISPR-Cas9-mediated deletion of *Stat5a* or *Stat5b* in HDM Der p1-specific 1DER TCR-transgenic CD4^+^ T cells results in impaired differentiation of GATA3^+^ Th2 cells and a reciprocal increase in Tfh cells in the mediastinal lymph node (LN), with more pronounced impact by the deletion of *Stat5b* than *Stat5a* (9). Thus, the IL-2–pSTAT5 signaling axis triggers the IL-4–GATA3 positive feedback loop in CD4^+^ T cells together with c-Maf and collectively promotes and stabilizes the Th2 lineage commitment.

#### 2.2.2 IL-2 in non-Th2 effector CD4^+^ T cell differentiation

While the abovementioned studies underscore the importance of IL-2 in Th2 cell differentiation, other studies found its role in Th1 cell differentiation, making the role of IL-2 as a specific fate-imprinting factor for Th2 cell differentiation unclear. Under Th1-polarizing conditions *in vitro*, pSTAT5 promotes the expression of Th1-associated genes, including *Il12rb*, *Tbx21*, and *Ifng* (18, 31). Consistently, during lymphocytic choriomeningitis virus (LCMV) infection *in vivo*, genetic ablation or shRNA-mediated knockdown of CD25 in CD4^+^ T cells results in a reduction of Th1 cell frequency in antigen-specific CD4^+^ T cells (16, 17). Likewise, in mixed bone marrow (BM) chimeric mice reconstituted with wild-type (WT) and *Il2ra* (CD25)*^−/−^* BM cells, infection with *Listeria monocytogenes* results in a reduced frequency of Th1 cells in the *Il2ra^−/−^* CD4^+^ T cells compared to the WT counterpart (15). Notably, in all of these cases, the reduction of Th1 cells is accompanied by a significant increase in Tfh cell differentiation, consistent with the well-supported role of IL-2 in suppressing Bcl6, the master transcription regulator for Tfh cell differentiation (32). Taken together, these studies indicate that IL-2 skews CD4^+^ T cell differentiation toward Th1 while suppressing Tfh cell development under the Th1-skewing immune environment.

#### 2.2.3 Differential requirement of IL-2 in Th1 and Th2 cell differentiation

Recombinant IL-2 is commonly used as a CD4^+^ T cell culture supplement to keep CD4^+^ T cells proliferating without skewing them to the Th1 or Th2 state. Studies discussed above have demonstrated the requirement of IL-2 for the differentiation of both Th1 and Th2 cells under respective conditions, but they did not directly address if IL-2 preferentially induce one state over the other (8–18). In contrast, the suppression of Tfh differentiation by IL-2 has been generally observed under both Th1- and Th2-biased conditions (9, 14–16, 21). Does this mean that IL-2 only plays a role in non-Tfh–Tfh bifurcation, or does it also play a role in Th2-specific lineage commitment?

To address this question, we recently performed experiments by adoptively transferring equal numbers of congenically labeled *Il2ra^+/+^* (WT) and *Il2ra^+/−^* or WT and *Il2ra^−/−^* ovalbumin (OVA)-specific OT-II TCR-transgenic CD4^+^ T cells into WT recipients immunized with OVA plus papain (a Th2-biased adjuvant), Freund’s complete adjuvant (FCA, a Th1/Th2-mixed adjuvant), or CpG DNA (a Th1-biased adjuvant) in the footpad. These experiments showed a greater reduction in Th2 cells than in Th1 cells in CD25-deficient OT-II cells compared to the WT counterpart in the footpad-draining LN regardless whether the loss of CD25 was partial of complete. Notably, the partial loss of CD25 in the *Il2ra^+/−^* OT-II cells had no impact on their expansion, while the complete loss of CD25 significantly reduced their number. These data indicate that effector CD4^+^ T cell differentiation is tightly regulated by the IL-2R signaling in a dosage-dependent manner, and that Th2 cell differentiation is more stringently dependent on the IL-2R signaling than Th1 cells under these conditions (33).

### 2.3 Regulation of IL-2 availability during Th2 cell priming

#### 2.3.1 Local IL-2 availability during CD4^+^ T cell priming

If Th2 cell differentiation is more sensitive to IL-2 dosage than Th1 cells, how is the IL-2 availability regulated during Th2 cell priming? This is an interesting, and somewhat puzzling, question, as earlier studies found that Th2 cells are preferentially induced by weak TCR stimulation while strong TCR stimulation tends to drive Th1 cell differentiation, yet the amount of IL-2 production from the activated T cells generally correlates with the TCR signal strength (27, 34). One reason for the lack of IL-2 dosage sensitivity in Th1 cells is the direct inhibition of IL-2R signaling by strong TCR stimulation (27), but how is the optimal amount of IL-2 conveyed to Th2 cell precursors during priming, especially if they prefer weak TCR stimulation? It has been shown that IL-2 produced by T cells primed in the antigen-draining LN is rapidly soaked up by Tregs surrounding the primed T cells, effectively setting the threshold for self-tolerance, as any randomly activated self-reactive T cells that have escaped thymic selection would produce only mediocre amount of IL-2 that cannot overshoot the threshold (22–24). If so, how do the (weakly) activated Th2 cell precursors make IL-2 available to themselves?

#### 2.3.2 IL-2 enrichment at the interface of homotypic T–T cell interactions

Upon TCR stimulation both *in vivo* and *in vitro*, CD4^+^ T cells have been shown to form homotypic T–T cell clusters with multifocal synapses, with IL-2 specifically enriched at the cell-cell interface. At the T–T cell interface, pSTAT5 forms puncta facing neighboring T cells, suggesting localized IL-2R signaling in the cluster (35). While such clustering itself does not seem to be specific to Th2 cell priming (35), Szeto et al. recently identified αvβ3 integrin as a key regulator of Th2 cell differentiation that facilitates homotypic T–T cell clustering (36). Integrin αv and β3 are upregulated directly by GATA3, and their specific deletion in T cells results in impaired T–T cell clustering and reduced IL-2-induced STAT5 phosphorylation following a stimulation with anti-CD3 and anti-CD28 mAbs *in vitro*. Notably, mice lacking integrin αv or β3 specifically in T cells show a reduction of Th2 cells in the mediastinal LNs upon challenge in the prime–challenge models of OVA/alum- and papain-induced pulmonary type 2 inflammation (36). Likewise, in mice immunized intranasally with HDM, He et al. recently demonstrated that HDM-specific 1DER CD4^+^ T cells expressing GATA3 form clusters around IL-2-expressing CD4^+^ T cells at the T–B cell border regions in the mediastinal LNs early after immunization. Importantly, 1DER cell-intrinsic CD25 is required for the expression of GATA3 and Blimp1, the latter of which further upregulates GATA3 and type 2 cytokine expression (9). These studies highlight the critical role of localized IL-2R signaling in the T–T cell clusters during Th2 cell differentiation.

#### 2.3.3 Role of DCs in T cell clustering during Th2 cell differentiation

Clustering of antigen-specific CD4^+^ T cells at the T–B cell border regions in the antigen-draining LN has also been observed in other Th2-biased conditions such as subcutaneous immunization with OVA and papain or alum and infection with a helminth parasite *Nippostrongylus brasiliensis* (37). Within these T cell clusters, GATA3^+^ Th2 cells co-express CD25 and show elevated pSTAT5 levels compared to GATA3*^−^* CD4^+^ T cells. Accordingly, blockade of IL-2 results in a reduction of GATA3^+^ IL-4^+^ Th2 cells without disrupting the clusters, indicating the role of IL-2 in Th2 cell differentiation but not cluster formation *per se* (37). However, given that antigen-specific naive CD4^+^ T cells are so rare that two independent clones are unlikely to be close to each other in the LN under physiological conditions, how is the formation of such clusters initiated *in vivo*? Blockade of CD28 or LFA-1, the T cell-expressed molecules critical for antigen-dependent DC–T cell interaction, but not genetic deletion of the LFA-1 ligand ICAM1 in antigen-specific CD4^+^ T cells, disrupts both T cell clustering and Th2 cell differentiation, suggesting a role for heterotypic DC–T cell interaction between T cell-intrinsic LFA-1 and DC-derived ICAM1 rather than T cell-derived ICAM1 in the initiation of T cell clusters (37).

Previous studies have shown that a specific subset of migratory conventional type 2 dendritic cells (cDC2s) expressing a C-type lectin CD301b (*Mgl2*) is selectively required for Th2 cell differentiation under these immunization conditions (38, 39). Accordingly, mice lacking IRF4 specifically in DCs (Irf4^ΔDC^ mice) fail to develop Th2 cells upon subcutaneous immunization with OVA plus papain or infection with *N.brasiliensis*, as those mice have reduced CD301b^+^ cDC2s in the skin-draining LNs due to their migration defect (40, 41). Moreover, direct antigen presentation by CD301b^+^ cDC2s is required for the differentiation of antigen-specific Th2 cells (33). CD301b^+^ cDC2s are specifically localized at the T–B cell border regions near the high endothelial venules in the LNs and scan the antigen specificity of incoming naïve CD4^+^ T cells (42, 43). Importantly, Lyons-Cohen et al. demonstrated that the Th2-initiating “macro-clusters”, marked by dense aggregates of proliferating CD4^+^ T cells that express IL-4, GATA3, and IRF4, are surrounded by CD301b^+^ DCs and are reduced in the Irf4^ΔDC^ mice (37). Taken together, these studies suggest that CD301b^+^ DCs play a role in the formation of antigen-specific CD4^+^ T cell clusters that support their subsequent differentiation into Th2 cells.

#### 2.3.4 Role of DC-derived IL-2 in Th2 cell fate instruction

Paracrine, but not autocrine, IL-2 from neighboring T cells has been considered as the main source of IL-2 for differentiating effector CD4^+^ T cells (44–46), presumably because the IL-2 expression is suppressed by the IL-2R signaling (5, 6). DiToro et al. (46) showed that activated CD4^+^ T cells rapidly bifurcate into IL-2^+^ and IL-2^−^ pSTAT5^+^ populations, which subsequently become Tfh and non-Tfh effector cells, respectively. Formation of activated CD4^+^ T cell clusters likely facilitates their access to IL-2 locally produced by the neighboring T cells and accelerate non-Tfh–Tfh bifurcation, but it leaves the question open as to how such IL-2-dependent activation mechanism is initiated, because a rare, single antigen-specific CD4^+^ T cell alone would not be able to form a cluster until they proliferate to a certain level.

**Figure 1:**
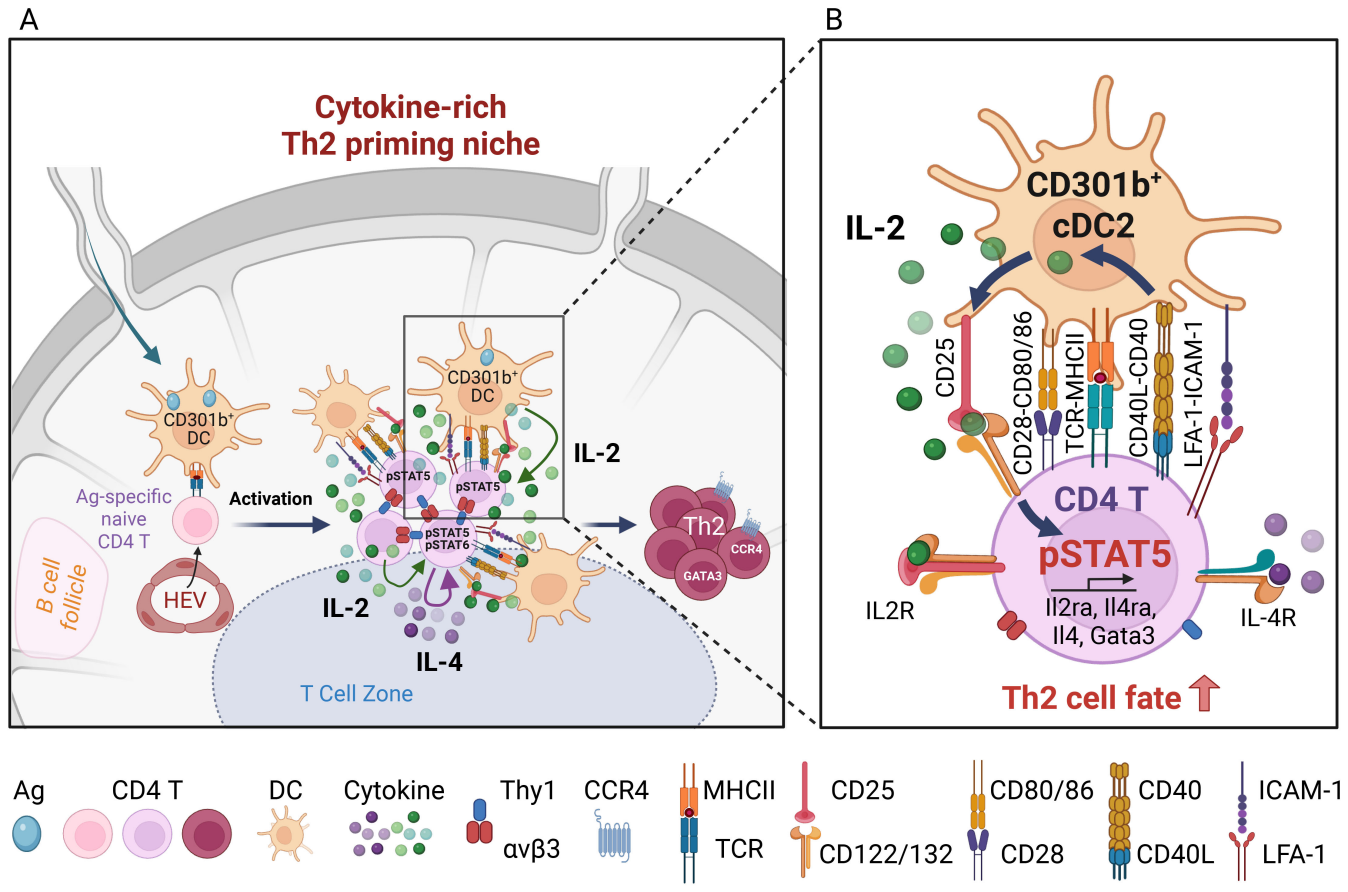
Role of CD301b^+^ DC-derived IL-2 in Th2 cell fate instruction. **(A)** Upon reaching the LN, skin-derived migratory CD301b^+^ DCs localize to the T-B boundary regions near the high endothelial venules (HEVs), where they interact with incoming naïve CD4^+^ T cells and provide cognate stimulation. Activated CD4^+^ T cells form T-T aggregates and organize into macro-clusters. These macro-clusters facilitate close interactions among CD4^+^ T cells via integrin αvβ3-Thy1 binding, and between CD4^+^ T cells and CD301b^+^ cDC2s through ICAM-1-LFA-1 interactions and co-stimulatory molecules such as CD40, CD80, and CD86. These clusters create a microenvironment that is enriched for T cell- and CD301b^+^ DC-derived IL-2, as well as T cell-derived IL-4. **(B)** Upon cognate interaction with antigen-specific CD4^+^ T cells, CD40 ligation on CD301b^+^ DCs enhances DC-intrinsic IL-2 production, which is required for maximal CD25 expression on the cognate CD4^+^ T cells. Maximal IL-2R signaling in CD4^+^ T cells is specifically required for Th2 fate commitment. CD301b^+^ cDC2-intrinsic CD25 facilitates the directed action of IL-2 toward cognate CD4^+^ T cells to support their Th2 fate decision, while limiting IL-2 access to nearby Tregs. Image created with Biorender.com.

In addition to activated CD4^+^ T cells, DCs can also produce IL-2 upon CD40 ligation or stimulation with IL-33 (47–50). Interestingly, previous studies found that DCs stimulated with CD40 ligand or IL-33 induce Th2 cell differentiation (51–53). In addition, particulate adjuvants like alum induce IL-2 production in DCs via the Syk-calcineurin-NFAT signaling axis, which is required for alum-induced antigen-specific CD4^+^ T cell proliferation and the generation of antigen-specific IgG1 and IgE (54). We recently showed that CD40 expression in CD301b^+^ cDC2s is specifically required for papain-induced Th2 cell differentiation (33). Stimulation of CD40 *in vivo* results in IL-2 production specifically from CD301b^+^ cDC2s, which is critical for optimal IL-2 receptor signaling and Th2 differentiation in antigen-specific CD4^+^ T cells in the antigen-draining LN. Mice lacking either MHCII or IL-2 specifically in CD301b^+^ cDC2s exhibit reduced CD25-STAT5 signaling in antigen-specific CD4^+^ T cells and impaired Th2 cell differentiation without affecting Th1 cells while increasing Bcl6^+^ PD-1^+^ Tfh precursors (33). Importantly, CD40 stimulation upregulates not only IL-2 production but also CD25 expression specifically in CD301b^+^ cDC2s, and genetic deletion of CD25 in CD301b^+^ cells results in a Th2-specific differentiation defect (33). However, CD301b^+^ cDC2-intrinsic CD25 does not seem to induce STAT5 phosphorylation in CD301b^+^ cDC2s due to the lack of CD122 (IL-2Rβ), but it is instead required for the maximal activation of STAT5 in the cognate CD4^+^ T cells but not Tregs in the same LN (33). These data indicate that CD301b^+^ cDC2 utilize their own CD25 to facilitate the directed action of IL-2 toward the cognate CD4^+^ T cells to support their Th2 differentiation, effectively blocking it from being consumed by surrounding Tregs (**Figure 1**).

#### 2.3.5 Role of B cell-derived IL-2 in Th2 cell differentiation

Like activated DCs, earlier studies found IL-2 production from B cells activated *in vitro* (55, 56). For instance, B cells co-cultured with Th2 cells produce IL-2 along with IL-4 and IL-13, whereas those co-cultured with Th1 cells produce IL-2 along with IFNγ and IL-12 (57). Critical role of B cell-derived IL-2 in Th2 cell differentiation was demonstrated in a mouse infection model with *Heligmosomoides polygyrus*, a strictly enteric helminth parasite. B cell-intrinsic MHCII, IL-4Rα, and IL-2, as well as parasite-specific antibodies are all required for parasite clearance upon secondary infection. Notably, BM chimeric mice lacking IL-2 specifically in B cells show a significant reduction in Th2 cells and parasite-specific antibody titers, suggesting that B cell-derived IL-2 is a critical regulator for Th2 cell differentiation (58). However, it remains unclear if B cell-derived IL-2 is critical for the Th2 fate decision early in priming or for amplification of Th2 cells later during their expansion.

## 3 Role of IL-2 in type 2 immune effector cells

IL-2 has traditionally been seen as the driver of expansion and maintenance of antigen-specific T cells during priming and rechallenge (59), but recent studies revealed the role of IL-2 in regulating tissue-resident lymphocytes such as CD4^+^ tissue-resident memory T (Trm) cells, Tregs and type 2 innate lymphocytes (ILC2s) in the context of type 2 immunity.

### 3.1 Role of IL-2 in regulating Th2 tissue-resident memory cells

In addition to its role in Th2 cell fate determination during priming, IL-2R signaling has been shown to be essential for directing the migration and residency of Th2 effector cells in the lung during allergic responses (60). In mixed BM chimeric mice reconstituted with WT and *Il2ra^−/−^* BM cells, intranasal sensitization and challenge with HDM results in comparable expansion of HDM Der p1-specific T cells in lymphoid organs. However, unlike their wild-type counterpart, *Il2ra^−/−^* T cells fail to accumulate in the lungs to form Trm cells, likely due to reduced expression of chemokine receptors CCR4 and CXCR3 and increased expression of CD69, which leads to their retention within the LNs (60). These data suggest a critical role of CD4^+^ T cell-intrinsic IL-2R signaling in the formation of lung Th2 Trm in allergic inflammation, though the critical source of IL-2 remains unclear.

### 3.2 Role of IL-2 in regulating ILC2s during type 2 inflammation

ILC2s are a major source of type 2 cytokines in the peripheral tissues and thereby play critical roles in type 2 inflammation and metabolic homeostasis (61). IL-2 is not required for the development of ILC2s, but it promotes their proliferation and production of type 2 cytokines such as IL-5, IL-9 and IL-13 during type 2 immune responses (62–66). In *Rag1^−/−^* and *Rag2^−/−^* mice, which lack T and B cells, treatment with IL-2 complexed with anti-IL-2 mAb (IL-2c) leads to a robust expansion and activation of ILC2s across different organs including the skin, lung, and intestine and boosts production of IL-5 and IL-13 (63, 64, 67). Those IL-2c-expanded ILC2s induce eosinophilic inflammation in the lungs and skin and confer protection against *N.brasiliensis* and *H.polygyrus* by reducing worm burdens (62–64). Additionally, IL-2, particularly in combination with IL-25 or IL-33, induces a cholinergic phenotype in ILC2s by upregulating choline acetyltransferase (ChAT), which is required for optimal proliferation and cytokine production by ILC2s and host protection against Nb infection (68). Together, these studies demonstrate that IL-2 enhances innate type 2 immune responses independent of adaptive immunity by promoting ILC2 effector function through cytokine and acetylcholine pathways.

### 3.3 Sources of IL-2 in regulating ILC2s

ILC2s are predominantly tissue-resident cells (69–72), suggesting that their activation by IL-2 requires local source of this cytokine. For instance, coculturing CD4^+^ T cells and ILC2s induces type 2 cytokines and ILC2 proliferation in an antigen- and MHCII-dependent manner, while neutralizing IL-2 in the CD4^+^ T cell–ILC2 coculture results in abrogated type 2 cytokine production from ILC2s, suggesting that Th2 cell-derived IL-2 plays a key role in activating ILC2s in the peripheral tissue (62). Accordingly, ILC2-intrinsic MHCII expression has been shown to be required for the capacity of ILC2s to expel Nb (62).

In addition to Th2 cells, B cell-derived IL-2 can also induce expansion of ILC2s. In mice expressing Cre-inducible IL-2 expression in B cells, ILC2s increase 100-fold in the spleen and mediate IL-5-driven eosinophilic expansion while mice with Cre-inducible IL-2 in T cells do not show this phenotype (73), presumably because of the close proximity of ILC2s to B cell follicles in lymphoid organs (74).

Lastly, mast cell-derived IL-2 has also been shown to regulate ILC2s. Like ILC2s, mast cells are tissue-resident cells and play a critical role in type 2 inflammation, most notably in IgE-mediated hypersensitivity. However, in the context of *Aspergillus fumigatus* infection and cystic fibrosis, IL-9-stimulated mast cells produce IL-2, promoting the expansion of CD25^+^ ILC2s and Th9 cells, thereby amplifying eosinophilic inflammation and airway pathology (75). It is however important to note that mast cell-derived IL-2 has been shown to play an anti-inflammatory role by promoting Treg cell expansion in other models of allergic inflammation such as oxazolone-induced dermatitis and papain-induced airway hypersensitivity (76–78).

Collectively, these studies show an important role of tissue-resident source of IL-2 in regulating type 2 inflammation through modulating the activation of ILC2s.

## 4 Concluding remarks

Although IL-2 has often been seen as a “neutral” driver of T cell proliferation, the studies discussed above underscore its specific role in type 2 immunity. Some of these studies highlight the importance of quantitative regulation of IL-2R signaling (e.g., CD25 gene dosage, proximity to the IL-2 source) in the qualitative outcome such as Th2 differentiation and ILC2 activation, indicating that fine-tuning of IL-2R signaling is crucial for the immune homeostasis. However, our understanding of the mechanisms governing such fine-tuning of IL-2R signaling is still incomplete. For example, while we now know that CD301b^+^ cDC2-derived IL-2 is critical for full upregulation of IL-2R signaling in the cognate CD4^+^ T cells (33), it is unclear why the activated CD4^+^ T cell-derived IL-2 is insufficient for Th2 cell differentiation. Insights into these mechanisms may enable spatially targeted immunotherapies for type 2 inflammation-associated diseases such as asthma, allergy, and helminth infections.
